# Risk Matrix for Violent Radicalization: A Machine Learning Approach

**DOI:** 10.3389/fpsyg.2022.745608

**Published:** 2022-05-12

**Authors:** Krisztián Ivaskevics, József Haller

**Affiliations:** Department of Criminal Psychology, Faculty of Law Enforcement, University of Public Service, Budapest, Hungary

**Keywords:** machine learning, terrorism, violent extremism, risk assessment, XGBoost

## Abstract

Hypothesis-driven approaches identified important characteristics that differentiate violent from non-violent radicals. However, they produced a mosaic of explanations as they investigated a restricted number of preselected variables. Here we analyzed without a priory assumption all the variables of the “Profiles of Individual Radicalization in the United States” database by a machine learning approach. Out of the 79 variables considered, 19 proved critical, and predicted the emergence of violence with an accuracy of 86.3%. Typically, violent extremists came from criminal but not radical backgrounds and were radicalized in late stages of their life. They were followers in terrorist groups, sought training, and were radicalized by social media. They belonged to low social strata and had problematic social relations. By contrast, non-violent but still criminal extremists were characterized by a family tradition of radicalism without having criminal backgrounds, belonged to higher social strata, were leaders in terrorist organizations, and backed terrorism by supporting activities. Violence was also promoted by anti-gay, Sunni Islam and Far Right, and hindered by Far Left, Anti-abortion, Animal Rights and Environment ideologies. Critical characteristics were used to elaborate a risk-matrix, which may be used to predict violence risk at individual level.

## Introduction

Research into terrorism can be divided into several major areas. One approach views radicalization as a process (Borum, 2011) and addresses the pathways that lead to extremism in general and violent extremism by investigating the life history of individuals in the light of underlying individual characteristics, life events and social conditions (Borum, 2003; Moghaddam, 2005; McCauley and Moskalenko, 2008; Aghabi et al., 2017). The aim of this approach is to understand the process of radicalization, which may reveal opportunities for interrupting or reversing its progression. The delineation of terrorist typologies—the second popular approach—is mainly based on structured professional judgments (Pressman and Flockton, 2012; Sarma, 2017; Meloy, 2018), which was indicated as highly preferable by scholars of the field (Monahan, 2012). The third major approach aims at the identification of risk factors which can be used in counterterrorism activities (Pressman and Flockton, 2012; Cook, 2014; Lloyd and Dean, 2015; Meloy, 2018). Ideally, this third approach—adopted in this study—would serve as an empirical basis for the other approaches and may provide means to improve both our views on radicalization pathways and expert opinion-based terrorist typologies.

It was suggested that risk assessment should have four components “(1) identifying empirically-valid risk factors, (2) determining a method for measuring (scoring) risk factors, (3) establishing a procedure for combining scores on the risk factors, and (4) producing an estimate of violence risk” (Skeem and Monahan, 2011). However, the identification of empirically valid risk factors was not accomplished fully so far. Thus, the need of identifying such factors is still current. Attempts to reach the goal can roughly be grouped into two major categories: hypothesis-driven and mathematics-driven approaches.

Hypothesis-driven approaches borrowed concepts from various scientific disciplines to select critical factors that may underlie violent radicalization (LaFree et al., 2018). Such studies used concepts of communication science (Youngblood, 2020), criminology (LaFree et al., 2018, 2019), economics (Varaine, 2019), political science (Abrahms, 2012), social psychology (Jasko et al., 2017; Carson et al., 2019), sociology (Becker, 2019; Ligon et al., 2019), etc. Typically, studies start by a thorough analysis of the theoretical explanations of violence, which serve as criteria for the selection of the variables chosen for analysis. The main statistical tools employed were bivariate correlations, often supplemented with multivariate approaches. Those who pursued this route were regularly focusing on differences between violent and non-violent extremists, and preferentially relied on the “Profiles of Individual Radicalization in the United States (PIRUS)” database developed by the National Consortium for the Study of Terrorism and Responses to Terrorism (START), University of Maryland (Jensen et al., 2015).

The main advantage of this approach is its solid theoretical framework, which helps avoiding the statistical pitfalls of “fishing expeditions.” At the same time, however, such studies deal with a relatively low number of factors, i.e., those relevant for the theoretical framework addressed. For instance, three studies addressed violent extremism based on 23, 29, and 21 variables (Jasko et al., 2017; LaFree et al., 2018; Becker, 2019). Albeit these numbers were by far not negligible, they constituted about one fifth of the number of factors contained in the PIRUS database from where data were extracted. More importantly, the factors showed little overlap across studies due to the differential theoretical approach. For instance, Becker (2019)— who focused on social control and social learning theories—included only 30% of the factors studied by LaFree et al. (2018) who employed a criminological approach. On its turn, Jasko et al. (2017)—who addressed the psychological concept of “quest for significance”—studied only 38% of the factors that were also studied by LaFree et al. (2018). Naturally, different theoretical approaches ask for different sets of factors; yet hypothesis-based approaches still provide a fragmented picture of the factors that engender violence. It is also worth to note that the context of the investigation influences the significance of interactions, especially in the case of multivariate analyses, where the role of a particular factor may be made conspicuous or may be masked by the factors investigated concurrently. For instance, marital status influenced violence in two but failed to influence it in the third study referred to above. Likewise, criminal history had a significant impact on violence in only one of the three studies.

Overall, theory-based approaches that employed the PIRUS database made important contributions to understanding terrorism. They helped understand motivations (Jasko et al., 2017), outlined criminological concomitants (LaFree et al., 2018), revealed the role of social control and social learning (Becker, 2019), helped understanding interactions between social phenomena and individual factors (Youngblood, 2020), allowed the description of five terrorist types (Ligon et al., 2019), revealed differences between terrorist ideologies (Jensen et al., 2015; Freilich et al., 2018; Varaine, 2019), outlined the role of prison radicalization (LaFree et al., 2019), revealed factors affecting plot success (Austin, 2019), and uncovered differences between criminal and terrorist groups (Pyrooz et al., 2017). Despite important achievements, however, hypothesis-driven approaches provide a mosaic of explanations rather than a unitary system, for which their predictions seem incomplete. More precise predictions may be achieved by mathematical approaches.

The basic assumption of the mathematical approach is that the features of terrorists and their organizations are organized into patterns that can be described mathematically and used for prediction. Instead of relying on hypotheses, researchers employ data mining techniques to extract hidden predictive knowledge without *a priori* assumptions (Gassebner and Luechinger, 2011; Tolan and Soliman, 2015; Kerodal et al., 2016; Basuchoudhary and Bang, 2018).

Mathematical approaches include the development of equations that may describe, for instance the spreading and amplification of online support for terrorism (Johnson et al., 2016) or predict the group responsible for a terrorist attack (Sachan and Roy, 2012). In recent years, however, machine learning algorithms seem to take the lead in this field. They automatically evaluate the relationships between independent variables on a training dataset and apply the recognized rules to a test dataset to make predictions regarding the dependent variable. For instance, the algorithm may allocate individuals of the test dataset into violent and non-violent populations. The percent success rate of allocation indicates the degree of applicability of the procedure for predictions.

The preferentially employed database in this area was the Global Terrorism Database, also developed and maintained by START. The aims of studies was to predict: terrorism risk by analyzing internet communications (Cheong and Lee, 2011; Iskandar, 2017; Pelzer, 2018; Kalaiarasi et al., 2019), the location, timing and/or the type of future attacks (Ding et al., 2017; Verma et al., 2018; Hao et al., 2019; Singh et al., 2019; Uddin et al., 2020), the terrorist group responsible for an attack (Tolan and Soliman, 2015; Talreja et al., 2017; Alfatih et al., 2019), the weapons which may be used in forthcoming attacks (Verma et al., 2018; Narula et al., 2020) and global social determinants of terrorism (Gassebner and Luechinger, 2011). Two studies used the PIRUS database to identify Islamist radicals based on data unrelated to ideological backgrounds (Al-Zewairi and Naymat, 2017) and to predict the use of chemical/biological weapons (Guarrieri and Meisel, 2019). Prediction accuracy was around ∼80%.

Algorithms employed include decision trees (e.g., C4.5 algorithm), Deep Learning, Extreme Bound Analysis, Gradient Boosting, Iterative Dichotomizer, K-Nearest Neighbor, Maximum Likelihood Estimation, Naïve Bayes, Neural Network models, Random Forest, Rare Event Logit Model and Support Vector Machine. Although the identification of the “best” machine learning algorithm was evaluated in some of these papers, data remained inconsistent: the most accurate model in one study proved to be less accurate in another. The likely reason was that the accuracy of prediction depended to a great extent on the variables chosen for the model and the handling of missing data (Gruenewald and Pridemore, 2011; Tolan and Soliman, 2015).

Taken together, the mathematical approach lacks a theoretical background but seems to provide practically useful predictions. To our best knowledge, however, no study employed machine learning to differentiate violent from non-violent extremists so far.

Here we employed a machine learning approach to differentiate non-violent but still criminal extremists from those who were engaged in violent attacks. More conventional Multiple Regression models were also studied for comparison. We considered all extremists and all variables available in the PIRUS database without *a priori* assumption. Although a few variables were excluded, this was done exclusively for technical reasons as shown below. This approach deprived our analysis of the solid theoretical framework of hypothesis-driven approaches but offered the possibility of comparing the predictive value of variables that were usually not studied together as shown above. Similarly, all extremists were included in the analysis irrespective of their ideological background, which allowed the direct comparison of ideologies regarding their potential to generate violence.

The primary aim of the study was to identify those variables and individual characteristics that predicted the emergence of violence in extremists, and to evaluate their predictive power. The secondary aim was to evaluate the relative importance of individual characteristics, by producing a rank order, which may help differentiate more from less important but still predictive characteristics. Finally, we aimed at generating a matrix of proviolence and antiviolence characteristics, which may be used in the future for risk assessment purposes. Ideally, one could find the place of an extremist within the matrix based on his/her characteristics and predict violence risk based on this place.

## Materials and Methods

### The Database Investigated

Data were drawn from the public release version of the PIRUS 2018 database (START, 2018). This is freely available at https://www.start.umd.edu/profiles-individual-radicalization-united-states-pirus-keshif, and was created by 15 members of the PIRUS group listed on the PIRUS homepage.^1^ The database was based on open-sources and extant START research products. The preliminary list contained 4,000 individuals from which the final list of 2,148 was created by the evaluation of inclusion requirements (see below) by full-time project researchers, or trained research assistants whose evaluations were reviewed by full-time project researchers. In the third stage, researchers coded the relevant background, contextual, and ideological information. Random sampling techniques were used to maximize the representativeness of the dataset at all points in time that were covered by the project (START, 2018).

The database contains information on individuals who (1) were fully or partially radicalized in the United States, (2) espoused ideological motives, (3) engaged in ideologically motivated acts, and (4) were either arrested, indicted, or killed because of their ideological activities in the United States (Jensen et al., 2016). Individuals fulfilling the first three criteria were also included if they were members of a designated terrorist organization or were associated with an organization whose leaders or founders were indicted of an ideologically motivated violent offense. Consequently, neither foreign fighters nor United States extremists active abroad were included in the database. The version used in this study contained information on 2,148 extremists characterized by 112 variables (called “fields” in the codebook of the database). These were arranged by the authors of the database into groups of variables which they clustered into six “supergroups” (Table 1). The variables were chosen to represent the radicalization processes as proposed by five core theories, e.g., cost/benefit theory, psychological models, recruitment theory, social identity theory, and social movement theory (Jensen et al., 2016). Each variable could take several values, which represented terrorist characteristics. For instance, the variable “Broad ethnicity” covered the following seven characteristics in the database: Hispanic/Latino, Black/African American, White, Middle Eastern/North African, Native American, Asian, and Other. As all variables covered several characteristics, the individual contribution of these were evaluated after the identification of critical variables.

**TABLE 1:** The structure of, and terrorist characteristics in, the PIRUS 2018 Database.

Variable groups	Variables (“fields”)	Terrorist characteristics (values)
**Plot and Consequences (“super-group” 1)**		
This super-group of variables were directly indicative of the dependent variable “Violence”; consequently, it was not considered in analysis and was omitted from this table.		
**Group Nature (“super-group” 2)**		
Group details	Group_membership	(0) Not member (1) Informal group (2) Formal extremist group (3) Above-ground group
	Terrorist_Group_Name	Not outcome measure
Recruitment details	Actively_Recruited	(0) No (1) Yes
	Recruiter	Not outcome measure
	Actively_Connect	(0) No (1) Prior to (2) After radical behaviors
Group activities and dynamics	Group_Competition	(0) No (1) Yes
	Role_Group	(0) Loose Associate (1) Follower (2) Leader
	Length_Group	Month in group
	Clique	(0) No (1) Yes
	Clique_Radicalize	Not outcome measures
	Clique_Connect	
**Radicalization (“super-group” 3)**		
Internet and media	Internet_Radicalization	(0) No (1) Some (2) Primary importance
	Media_Radicalization	
	Social_Media	
	Social_Media_Frequency	Not outcome measures
	Social_Media_Platform	
	Social_Media_Activities	
Radicalization ideology	Radicalization_Islamist	(0) No (1) Yes
	Radicalization_Far_Right	
	Radicalization_Far_Left	
	Radicalization_Single_Issue	
	Ideological_Sub_Category	(1) Militia/gun rights (2) White supremacist (3) Xenophobic (4) Anti-government (5) Christian Identity (6) Animal rights/Environmentalist (7) New Left (8) Black Nationalist (9) Anti-capitalist (10) Anarchist (11) Islamist (12) Puerto Rican nationalist (13) Irish Republican Army (14) Cult/idiosyncratic (15) Anti-abortion (16) Jewish Defense League (17) Anti-gay (18) Other (19) Male supremacist
Radicalization location and timing	Loc_Habitation	Not outcome measure
	Itinerant	(0) No (1) Yes
	External_Rad	
	Rad_Duration	Month radicalized
Extent of radicalization	Radical_Behaviors	(0) No (1) Associates with radicals (2) Changing lifestyle (3) Converting others (4) Distancing from past relationships (5) Legal activism (6) Material/financial support (7) Logistical support (8) Seeks training [(9) Active participation in plots (10) Active participation in violent plots]
	Radical_Beliefs	(0) No evidence (1) Exposure to (2) Pursues information (3) Full knowledge of tenets (4) Shares beliefs (5) Deep commitment
Radicalizing events	US_Govt_Leader	(0) No (1) Yes
	Foreign_Govt_Leader	
	Event_Influence	(0) None (1) September 11 attacks (2) Vietnam War (3) Cold War (4) First Gulf War (5) Afghanistan/Iraq War (6) Ruby Ridge (7) Arab Spring (8) Other
Radicalization process	Beliefs_Trajectory	(1) Gradual (2) Key moments
	Behaviors_Trajectory	
	Radicalization_Sequence	(1) Beliefs preceded radical behaviors (2) Beliefs followed radical behaviors (3) Concomitant
Radicalizing sites	Radicalization_Place	(0) No radicalization (1) Place of worship (2) Educational institution (3) Social club
	Prison_Radicalize	(0) Full radicalization before prison (1) Increased in prison (2) Maximum after prison (3) Maximum in prison
**Demographics (“super-group” 4)**		
General details	Broad_Ethnicity	(1) Hispanic/Latino (2) Black/African-American (3) White (4) Middle Eastern/North African (5) Native American (6) Asian (7) Other
	Age	Years of age
	Marital_Status	(1) Single (2) Married (3) Divorced or Separated (4) Widowed
	Children	No. of children
	Age_Child	Not outcome measure
	Gender	(1) Female (2) Male
Religious background	Religious_ Background	(1) Sunni (2) Shi’a (3) Sufi (4) Other (5) Unspecified Islam (6) Evangelical Protestant (7) Mainline Protestant (8) Catholic (9) Orthodox (10) Other (11) Unspecified Christianity (12) Jewish (13) Buddhist (14) Hindu (15) New religion (16) Agnostic (17) Atheist (18) Other
	Convert	(0) No (1) Prior to (2) During (3) After radicalization
	Convert_Date	Not outcome measure
	Reawakening	(0) No (1) Prior to (2) During (3) After radicalization
	Reawakening_date	Not outcome measure
Citizenship history	Citizenship	Not outcome measure
	Residency_Status	(1) Born Citizen (2) Naturalized Citizen (3) Permanent Resident (4) Temporary Resident (5) Undocumented resident
	Nativity	Not outcome measure
	Time_US_Months	Month in USE
	Immigrant_Generation	(0) 3 + (1) First (2) Second
	Immigrant_Source	Not outcome measure
Ties to society	Language_English	(0) No (1) Yes
	Diaspora_Ties	(0) None (1) Weak (2) Strong
**Socioeconomic Status (“super-group” 5)**		
Education	Education	(1) No high school (2) Some High school (3) High school (4) Some College (5) College degree (6) Some vocational school (7) Vocational school degree (8) Some Master’s school (9) Master’s degree 1(0) Some Doctoral/Professional training (11) Doctoral/Professional degree
	Student	(0) No (1) Yes
	Education_Change	Not outcome variable
Finances and employment	Employment_Status	(1) Employed (2) Self-employed (3) Unemployed, seeking work (4) Unemployed, not seeking work (5) Student (6) Retired
	Change_Performance	(0) No (1) Yes
	Work_History	(1) Long-term Unemployed (2) Underemployed (3) Serially Employed (4) Regularly Employed
Military	Military	(0) No (1) Inactive, unknown deployment (2) Inactive, never deployed (3) Inactive, previously deployed (4) Active, unknown deployment (5) Active, never deployed (6) Active, deployed
	Foreign_Military	(0) No (1) Yes
Socioeconomic stratum	Social_Stratum_Childhood	(1) Low (2) Middle (3) High
	Social_Stratum_Adulthood	
	Aspirations	(0) No (1) Yes, no attempt to achieve (2) failed to achieve (3) achieved prior to radicalization
**Personal (“super-group” 6)**		
Abuse and psychological concerns	Abuse_Child	(0) No (1) By non-family (2) Family (3) Both
	Abuse_Adult	
	Abuse_Type	Not outcome variable
	Psychological	(0) No (1) Speculation (2) Diagnosed
	Alcohol_Drug	(0) No (1) Yes
Family and relationships	Absent_Parent	(0) No (1) Mother (2) Father (3) Both
	Overseas_Family	(0) No, (1) Yes
	Close_Family	(1) Distant (2) Close
	Family_Religiosity	(0) Secular (1) Somewhat (2) Very Religious
	Family_Ideology	(0) None (1) Islamist (2) Far right (3) Far left (4) Other (5) Single-Issue
	Family_Ideological_Level	Not outcome variable
	Prison_Family_Friend	(0) No (1) Yes
	Crime_Family_Friend	(0) No (1) Victim (2) Perpetrator (3) both
	Radical_Friend	(0) No (1) Yes, only legal activities (2) Non-violent illegal activities (3) Extremist violence
	Radical_Family	
	Radical_Signif_Othe	
	Relationship_Troubles	(0) No (1) Yes
	**Problematic Social Relations**	
	Unstructured_Time	
	Friendship_Source	(1) School (2) Work (3) Family (4) Religious group (5) Social Organization (6) Other
	Kicked_Out (marginalized)	(0) No (1) Yes
Criminal activity	Previous_Criminal_Activity	(0) No (1) Non-violent, minor (2) Non-violent, serious (3) Violent crime
	Previous_Criminal_Activity_Type	(1) Homicide (2) Rape (3) Robbery (4) Aggravated Assault (5) Burglary (6) Larceny-Theft (7) Motor Vehicle Theft (8) Arson (9) Simple Assault (10) Fraud (11) Forgery (12) Embezzlement (13) Driving under influence (14) Prostitution (15) Vandalism (16) Drug related (17) Parole violation (18) Firearm violation (19) Domestic violence (20) Other
	Previous_Criminal_Activity_Age	Not outcome variable
	Gang	(0) None (1) Street (2) Organized (3) Both
	Gang_Age_Joined	Not outcome variable
Mindset prior to radicalization	Trauma	(0) No (1) timing vis-à-vis radicalization unknown (2) Long before (3) Shortly before
	Other_Ideologies (prior radicalization)	(0) No (1) Yes
	Angry_US	
	Group_Grievance	(0) No (1) Yes, no personal connection (2) Personal connection (3) Direct experience
	Standing (diminution)	(0) No (1) Timing vis-à-vis radicalization unknown (2) Long before (3) Shortly before

*Variables (“Field Names”) were shown as in the database; characteristics (values) were abbreviated to fit table. Problematic social relations were depicted as platonic troubles in the database. Variables excluded from analysis were shown in small font. For other explanations see text.*

### Dependent and Independent Variables

The dependent variable was the field “Violent” of the database. This covered two characteristics: lack of violent activities (non-violent) and violent acts. The latter were defined in the Codebook as “actively participate in ideologically motivated operations/actions that resulted in causalities/injuries or clearly intended to result in causalities/injuries” (Codebook page 12). Thus, violent individuals perpetrated terrorist attacks that resulted in, or were clearly planned to result in injuries and/or fatalities. Noteworthy, non-violent extremists also engaged in unlawful activities by supporting extremist organizations and/or terrorist plots by, e.g., by providing financial or legal help or by recruiting new extremists. Thus, non/violent individuals were extremists who refrained from committing violent attacks but contributed to their completion. The variable “Violent” differentiated a non-violent (*N* = 908) and a violent group (*N* = 1240).

The remaining 111 variables were evaluated as predictors. Out of these, 32 were excluded for either of the following reasons: (1) The variable was directly indicative of violence. For instance, all variables included in the “Plot and Consequences” section described the plot, i.e., directly indicated whether the individual was violent; (2) data were missing for ≥85% of the sample. Missing data with the remaining 79 variables were handled by two different methodologies as shown below.

### Statistical Strategy

#### Multiple Regression and Bivariate Analyses

Multiple Regression analysis was performed by means of the Statistica software (TIBCO Software Inc, Palo Alto CA, United States). This extends regression to predict the value of the dependent variable based on the values of several predictor variables. Key measures include the coefficient of multiple correlation (Multiple R), F and *p* values indicative of the significance of prediction and the adjusted R-square. This indicates that percentage of variance in the dependent variable, which was explained by the independent variables. The latter is called prediction in statistical terms. The individual contribution of predictor variables to overall predictions is indicated by individual regression coefficients (β) and their t-statistics. The overall prediction by independent variables is usually attributed to those that have significant individual contributions.

**FIGURE 1:**
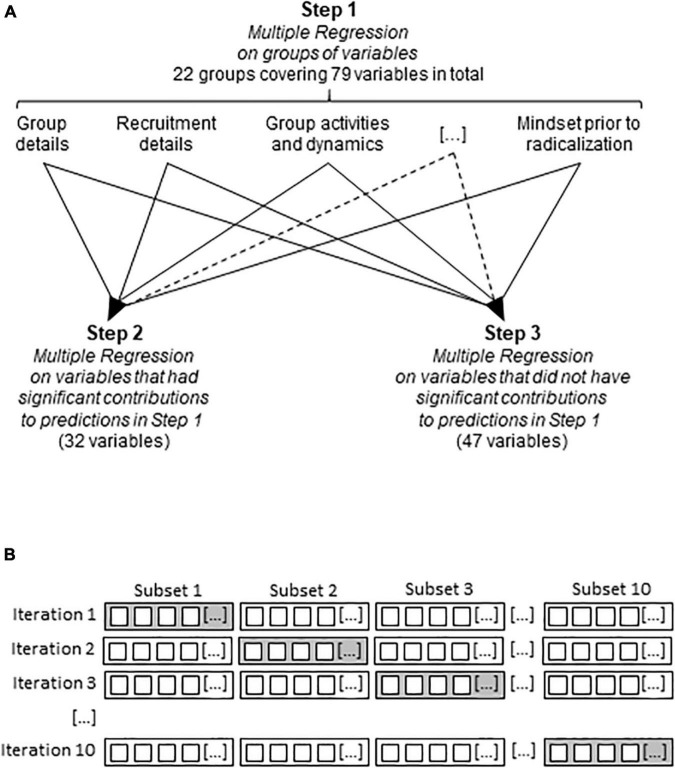
Strategy employed in Multiple Regression and machine learning analyses. **(A)** The stepwise approach employed in Multiple Regression. Note that the sample size did not allow the evaluation of all 79 variables in one single analysis, which explains the stepwise approach. […], variables not shown. **(B)** The partition of the database into training and testing data subsets with the XGBoost algorithm. The small squares represent the extremists included in the PIRUS database; the larger rectangles demarcate data subsets. White, training subset; gray, testing subset; […], data subsets not shown.

When all the variables were considered together, Multiple Regression could not be performed because the number of variables was too large for the sample size. Therefore, a three-step approach was adopted (Figure 1A). In Step 1, Multiple Regression was run separately on each group of variables contained in the database (see Table 1 for variable groups). Step 2 involved the repeating of the Multiple Regression analysis with those variables, which had a significant individual contribution to predictions in Step 1. In Step 3, Multiple Regression analysis was run with those variables, which did not have a significant contribution in Step 1. This analysis checked for the accuracy of variable selection in Step 1. Finally, the same analyses were performed after replacing missing data by mean substitution, a way offered by the Statistica software to address missing data.

To clarify the impact of individual characteristics covered by the variables, a bivariate *post hoc* comparison was performed (non-violent vs. violent). This was made by crosstabulation. To evaluate the extent of group differences, risk ratios were also calculated; this was the ratio of the prevalence of a characteristic in violent and non-violent individuals. Risk ratios above and below 1 indicated that the characteristic was more and less frequent, respectively, in violent as compared to non-violent individuals. Significance level was established at *p* < 0.05. Bonferroni adjustment was used to correct for errors deriving from multiple comparisons.

#### Machine Learning and Bivariate Analyses

The open-source ensemble machine learning algorithm XGBoost (eXtreme Gradient Boosting, 47) was used in the Python 3.6 programming interface. This was chosen for its outstanding performance in a wide range of classification and regression problems in various disciplines, and successful applications in scientific studies (Torlay et al., 2017; Aaboud et al., 2018). Training and testing datasets, i.e., those that enabled the algorithm to learn associations and to test predictions underwent 10 times repeated 10-fold cross-validation (James et al., 2013). This involves the division of the extremists of the PIRUS database into 10 equally sized subsets. In each iteration, nine subsets were used for training, whereas the 10th was used for testing. To account for the initial choice of the 10 partitions, the whole process was repeated 10 times by rotating the training and test datasets (Figure 1B). This procedure uses all observations for both training and testing and uses each for testing just once.

The individual contribution of predictor variables to the models was evaluated by their permutation importance (Strobl et al., 2008). This consists of calculating predictions after permutating the values of predictor variables one by one. The importance of a variable is inversely proportional to the decrease of prediction accuracy after the shuffling of its values (Molnar, 2019).

Albeit XGBoost was able to deal with all variables at the same time, analysis was performed in three steps, because during the optimization process (“boosting”) the algorithm keeps only one of those variables that correlate strongly, e.g., the one that has the highest permutation importance (predictive power). Consequently, the models contained three different sets of variables: (i) predictor variables that strongly predicted the dependent variable (permutation importance >1%); (ii) variables that were not selected because of their high correlation with the former, but which potentially may have become predictor variables if the former were missing from the database (predictor-like variables), and (iii) non-predictor variables. In Step 1, we allowed the algorithm to work with all the variables. In Step 2, we evaluated predictive power after excluding the predictor variables identified in Step 1 but keeping predictor-like and non-predictor variables. In Step 3, we included non- predictor variables. This approach answered two questions: (1) Can the most important variables be replaced by predictor-like variables, if they were missing from the database? and (2) Do variables with no permutation importance possess any predictive value?

Missing predictors were replaced by using Multiple Imputation with Chained Equations, with the mice package (van Buuren and Groothuis-Oudshoorn, 2010) of the R statistical software (R Development Core Team, 2013). Following the guidelines, different imputation methods were specified for different variable types: predictive mean matching (pmm) was used for continuous variables, logistic regression (logreg) for binary variables, polytomous logistic regression (polyreg) for unordered (nominal) categorical variables and proportional odds model (polr) for ordered (ordinal) categorical variables. A total of 20 imputed datasets were created over 20 iterations, which were then analyzed separately, and the results were pooled to obtain an overall estimation. The reported values for area under the curves (AUCs), precisions and recalls therefore represent the mean of each statistic across the 10 imputed datasets.

Performance measures were the Receiver Operating Characteristic Area Under the Curve (ROC-AUC) which represents the relationship between the probability of finding a true positive (an actual violent individual that was predicted violent by the model) and a false positive (a non-violent individual that was predicted violent by the model). The AUC limits for chance, acceptable, moderate, good, and excellent discrimination were 0.5–0.6, 0.61–0.7, 0.71–0.8, 0.81–0.9, and 0.91–1.0, respectively (Sjöstedt and Grann, 2002). We also included other metrics, e.g., precision, a measure of classification exactness and recall, a measure of classification completeness. In this case, 100% indicates perfect precision/recall, whereas 50% represents chance level. Finally, the importance of individual characteristics was also analyzed. This was done as described above.

## Results

### Critical Variables—Multiple Regression

In Step 1, we studied separately the predictive power of independent variables within variable groups. Out of the 22 such groups (see Table 1), 17 predicted violence significantly (Table 2). Out of the 79 variables belonging to these 17 groups, 32 variables had significant individual contributions to prediction (Table 2). Albeit predictions were significant, they explained a low share of variance in violence (∼4% on average).

**TABLE 2:** Explanations of variance in violence according to the multiple regression analysis.

STEP 1 Analysis by variable groups (see Table 1)			
Predictor variable groups (Variables with significant contributions)	Multiple R	F statistics	Variance explained
Family and relationships (*Family Ideology, Problematic Social Relations*)	0.404	*F*(13,161) = 2.42; *p* < 0.01	9.6%
Religious background (*Convert, Religious Background*)	0.322	*F*(3,319) = 12.31; *p* < 0.0001	9.5%
Extent of radicalization (*Radical Behaviors, Radical Beliefs*)	0.305	*F*(2,607) = 31.19; *p* < 0.0001	9.0%
Radicalization ideology (*Ideological Sub Category, Radicalization Far Left*, *Radicalization Far Right, Radicalization Islamist*)	0.293	*F*(4,2143) = 50.51; *p* < 0.0001	8.4%
Group nature (*Clique, Length Group, Role Group*)	0.250	*F*(7,625) = 5.96; *p* < 0.0001	5.2%
Socioeconomic stratum (*Aspirations*)	0.236	*F*(3,184) = 3.64; *p* < 0.05	4.1%
General details (demographics) (*Age, Broad Ethnicity, Gender*)	0.201	*F*(5,1070) = 9.03; *p* < 0.0001	3.6%
Radicalization location and timing (*External Radicalization, Radicalization duration*)	0.171	*F*(3,660) = 6.667; *p* < 0.005	2.5%
Criminal Activity (*Criminal Activity*)	0.166	*F*(3,1222) = 11.48; *p* < 0.0001	2.5%
Citizenship history (*Immigrant Generation, Time US Months*)	0.153	*F*(3,1630) = 13.04; *p* < 0.0001	2.1%
Internet and Media (*Media Radicalization*)	0.161	*F*(3,514) = 4.592; *p* < 0.005	2.0%
Radicalizing events (*Event Influence, Foreign Government Leader*)	0.154	*F*(3,856) = 6.93; *p* < 0.001	2.0%
Education (*Education*)	0.150	*F*(2,708) = 8.18; *p* < 0001	1.9%
Radicalizing sites (*Prison Radicalize*)	0.137	*F*(2,566) = 5.42; *p* < 0.005	1.5%
Abuse and Psychol. Concerns (*Alcohol/Drug abuse, Psychological*)	0.132	*F*(4,2143) = 9.50; *p* < 0.0001	1.5%
Finances and Employment (*Change Performance, Employment Status*, *Work History*)	0.115	*F*(3,2145) = 9.64; *p* < 0.0001	1.1%
Ties to society (*Diaspora Ties*)	0.093	*F*(2,1659) = 7.34; *p* < 0.001	0.7%
Radicalization process (*none*)	0.094	*F*(3,730) = 2.13; *p* < 0.1	None
Military (*none*)	0.031	*F*(2,1427) = 0.726; *p* < 0.5	None
Mindset prior to radicalization (*none*)	0.032	*F*(5,304) = 1.063; *p* < 0.4	None
*Average variance explained*	*3.95%*		

In Step 2, a Multiple Regression analysis was run on those 32 variables that had significant contributions in Step 1. For these variables, prediction power increased by one order of magnitude, indicating that violence was interactively predicted by variables belonging to the different groups of variables (Table 3). In Step 3, Multiple Regression was rerun on those 47 variables that had no significant contributions in Step 1. This combination of variables did not explain the variance in the dependent variable (Table 3). Thus, the selection of critical variables in Step 1 was adequate.

**TABLE 3:** Multiple Regression analysis of variables having or not having significant contributions to prediction in Step 1.

Original data	
Step 2 *Analyzed dataset:* Significant contributors in Step 1	Step 3 Non-significant contributors in Step 1
*Multiple R* = 0.841 *F*(10,102) = 24.61; *p* < 0.0001 *Variance explained*: 67.8% *Significant contributor variables in Step 2* Broad Ethnicity, Family Ideology, Ideological Sub Category, Media Radicalization, Previous Criminal Activity, Radical Behaviors, Radicalization Far Left, Radicalization Far Right, Religious Background	*Multiple R* = 0.342 *F*(32,165) = 0.687*; p* < 0.9 *Variance explained*: 0.0% *Significant contributors in Step 3* None
After mean substitution	
Step 2 *Analyzed dataset:* Significant contributors in Step 1	Step 3 Non-significant contributors in Step 1
*Multiple R* = 0.454 *F(22,2107)* = *13.36 p* < *0.0001* *Variance explained*: 19.1% *Significant contributor variables in Step 2* Broad Ethnicity, Family Ideology, Ideological Sub Category, Media Radicalization, Previous Criminal Activity, Radical Behaviors, Radicalization Far Left, Radicalization Far Right, Religious Background	*Multiple R* = 0.212 *F* (34,2114) = 0.81; *p* < 0.8 *Variance explained*: 0.0% *Significant contributor variables* None

*Note that Multiple Regression was run by activating the pairwise deletion and forward stepwise modes of function of the Statistica module, to minimize the impact of missing data.*

The mean substitution of missing data decreased the average prediction power to 1.9% in Step 1, and to 19.1% in Step 2 (Table 3). Thus, the interactions between variables were non-linear; consequently, mean substitution appeared inappropriate to control for missing data in this database.

### Critical Characteristics—Multiple Regression

**FIGURE 2:**
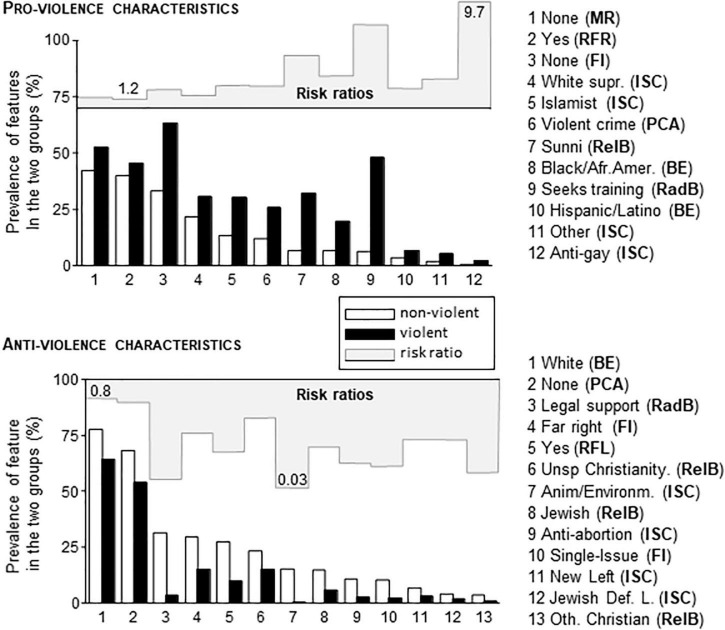
The share of various characteristics in non-violent and violent individuals of the PIRUS database. Characteristics were selected based on Multiple Regression analysis. The violent and non-violent groups were different in all cases after Bonferroni adjustment for repeated comparisons (*p* < 0.05 at least). Characteristic names (as shown in the database) were followed by the abbreviation of the variable where the characteristic belonged. BE, Broad ethnicity; FI, Family Ideology; ISC, Ideological Sub-Category; MR, Media Radicalization; PCA, Previous Criminal Activity; RadB, Radical Behaviors; RFL, Radicalization Far Left; RFR, Radicalization Far Right; RelB, Religious Background. Risk ratios were also shown as graphs on a scale of 1–10 (pro-violence characteristics) and 0–1 (anti-violence characteristics). The smallest and largest risk ratios were numerically shown as reference values.

Pairwise comparisons were performed within those variables that significantly predicted violence in Multiple Regression analysis Step 2. Only a subgroup of characteristics showed significant group differences (Figure 2). It also occurs that neither characteristic alone could predict violence. Some were rather frequent, which made them potentially useful, but these had low risk ratios. See, for instance, “none” (Media Radicalization) and “white” (Broad ethnicity) in Figure 2. With other characteristics, risk ratios were high yet their share in the population was too low to be used as individual predictor characteristics. See, for instance, anti-gay (Ideological Subcategory) and Other Christian (Religious background) in Figure 2. Therefore, we investigated in the followings how pro-violence and anti-violence characteristics were combined in individual extremists to see whether such combinations may be used for predictions.

**FIGURE 3:**
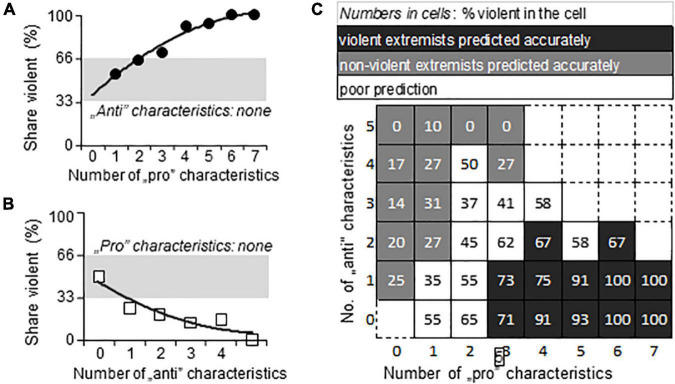
The prediction of violence by the number of pro- and anti-violence characteristics. **(A,B)** The dependence of the share of violent individuals on the number of pro-violence (“pro”) and anti-violence (“anti”) characteristics, respectively, when the opposite characteristic was not present in the individual. **(C)** The interactive dependence of violence on the relation between the number of pro-violence and anti-violence characteristics present in the individual. Gray, the area of chance prediction (34–66%, i.e., close to 50%).

The largest number of pro-violence and anti-violence characteristics concomitantly present in one and the same individual was 7 and 5, because alternative characteristics were mutually exclusive within variables (e.g., the individual could show only one of the seven characteristics covered by Broad Ethnicity). Although the maximum number of critical characteristics was already low compared to their relatively large number (25, see Figure 3), most individuals presented with even less critical characteristics namely with one or two. In addition, anti-violence and pro-violence characteristics were often present concomitantly in the same individual. As this resulted in a very high number of individual combinations, a characteristic-focused analysis could not be performed. It occurred, however, that the number of critical characteristics was relevant for predictions as shown below.

When no anti-violence characteristic was present in the individual (*N* = 384), the number of pro-violence characteristics predicted violence well (Figure 3A). Likewise, when anti-violence characteristics were unaccompanied by pro-violence ones (*N* = 227), the chance of violence was very low (Figure 3B). In most individuals, however, both categories of characteristics were present (*N* = 1137), and the share of violent individuals depended on the relationship between anti- and proviolence characteristics (Figure 3C). In about one third of the sample the analysis correctly identified violent individuals; in numerically the same number of cells non-violent individuals were identified correctly (in this case the chance of violence was low). In about one third of the cells, the prediction of violence was poor, i.e., was between 34 and 65%. The weighted mean of all these predictions was 61.2%, which was in line with the 66.7% explanation of variance by Multiple-Regression analysis of variables.

### Critical Variables—Machine Learning

The baseline XGBoost model that considered all the variables (Step 1) provided a final model that differentiated violent and non-violent extremists with good discrimination accuracy (Table 4). The 19 most important variables, i.e., those that had permutation importance larger than 1% contained 7 out of the 9 that were identified by Multiple Regression (Table 5). Moreover, most variables indicated as critical by the latter had high permutation importance according to machine learning. Nevertheless, XGBoost analysis revealed the importance of 12 variables that were not identified by Multiple Regression.

**TABLE 4:** Performance metrics of the XGBoost models with different predictor sets.

Original data			

Predictor dataset	ROC-AUC %	Precision %	Recall %
	M (SD)	M (SD)	M (SD)
All variables (79)	86.3 (2.3)	78.2 (3.5)	88.4 (3.0)
Predictor variables (19)	87.2 (2.2)	78.5 (3.6)	88.2 (3.0)
Predictor-like variables (?)	70.9 (3.5)	67.6 (4.3)	78.4 (4.0)
Not included in the model (?)	69.1 (3.7)	66.4 (4.4)	78.8 (3.9)

**After multiple imputation**			

**Predictor dataset**	**ROC-AUC %**	**Precision %**	**Recall %**
	**M (SD)**	**M (SD)**	**M (SD)**

All variables (79)	86.9 (3.2)	78.7 (7.5)	88.1 (4.3)
Predictor variables (19)	87.2 (3.0)	79.0 (7.3)	87.8 (4.7)
Predictor-like variables (?)	67.6 (5.9)	65.8 (10.1)	78.0 (8.1)
Not included in the model (?)	66.6 (5.9)	64.8 (11.4)	75.9 (9.6)

*M, mean; SD, standard deviation; ROC-AUC, Receiver Operating Characteristics – Area Under the Curve. Note ROC-AUC represents the discrimination accuracy of the models with 50% indicating chance level prediction and 100% indicating perfect prediction. Precision refers to the proportion of true violent extremists over the number of extremists who were predicted as violent. Recall refers to the proportion of true violent extremists over all actual violent extremists.*

**TABLE 5:** Average permutation importance of the 19 most important predictors listed in descending order.

Predictor variables	Variable group	Permutation importance (%)
*Radical Behaviors*	Radicalization	14.64
*Radicalization Far Left*	Radicalization	5.82
*Ideological Sub Category*	Radicalization	3.58
*Broad Ethnicity*	Demographics	1.25
Radical Friend	Personal	0.78
*Religious Background*	Demographics	0.54
Prison Radicalize	Radicalization	0.45
Radicalization Islamist	Radicalization	0.45
Group Membership	Group	0.35
*Radical Family*	Personal	0.35
Role Group	Group	0.33
Social Media	Radicalization	0.25
Convert	Personal	0.18
*Previous Criminal Activity*	Demographics	0.15
Problematic Social Relations	Personal	0.14
Radical Beliefs	Radicalization	0.13
Clique	Personal	0.11
*Media Radicalization*	Radicalization	0.10
Social Stratum Adulthood	Demographics	0.10

*Higher permutation importance values indicate stronger contribution to the prediction of the model. Underlined and italic, predictor variable also identified by Multiple Regression.*

Prediction slightly improved when the analysis was restricted to the 19 variables with permutation importance larger than 1%, suggesting that the removal of highly redundant variables increased the predictive accuracy of the model. In both cases, the recall of the models was considerably higher compared to precision, suggesting that the model predicted fewer false negatives (non-violent extremists who were in fact violent) than false positives (non-violent extremists predicted as violent). When only predictor-like and non-predictor variables were evaluated, all three, prediction power, precision and recall considerably decreased to acceptable/moderate performance. Finally, the elimination of both predictor and predictor-like variables reduced prediction accuracy considerably.

Note that data imputation did not affect performance notably (Table 4), which on one hand shows that the imputation method employed was able to correctly represent the non-linearity of interactions between variables but on the other hand it also shows that missing data did not affect prediction accuracies significantly.

### Critical Characteristics—Machine Learning

**FIGURE 4:**
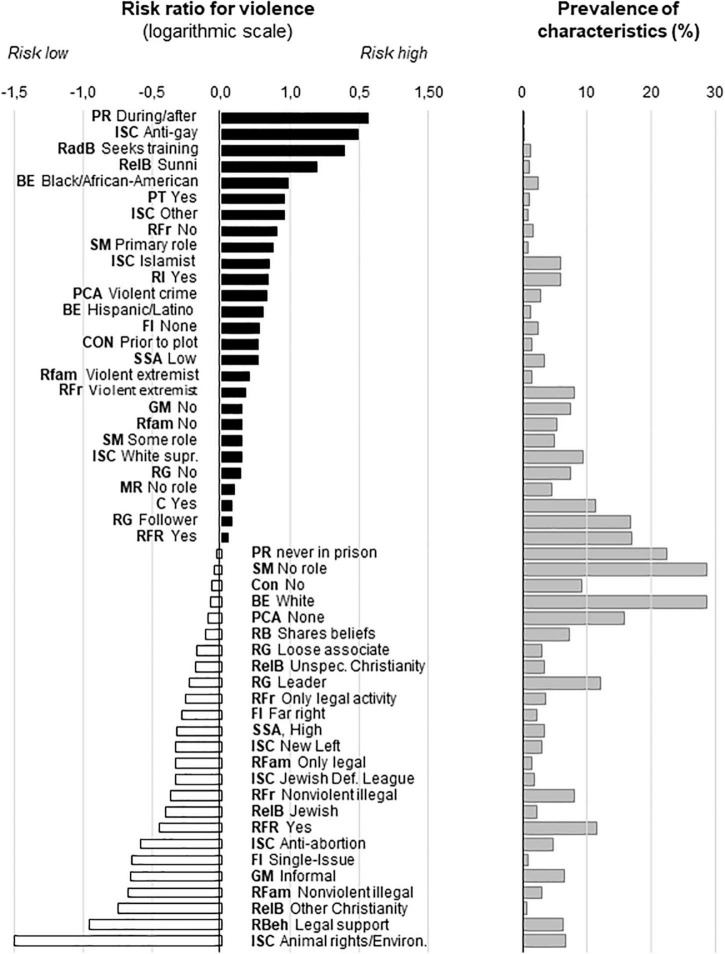
Critical individual characteristics identified by machine learning and their share in the population. The names of variables were abbreviated (bold); those of characteristics were shortened to fit figure. The share of the characteristic was shown for the non-violent group. Abbreviations for variables. BE, Broad ethnicity; C, Clique; Con, Convert; FI, Family Ideology; GM, Group Membership; ISC, Ideological Sub Category; MR, Media Radicalization; PCA, Previous Criminal Activity; PR, Prison Radicalize; PSR, Problematic Social Relations; RadB, Radical Behaviors; RB, Radical Beliefs; RBeh, Radical Behaviors; RelB, Religious Background; RFam, Radical Family; RFL, Radicalization Far Left; RFr, Radical friend; RFR, Radicalization Far Right; RG, Role Group; RI, Radicalization Islamist; SM, Social Media; SSA, Social Stratum Adulthood.

The 19 most important variables covered in total 113 characteristics, out of which 53 showed significant group differences (χ^2^ was between 5.85 and 180.62; corresponding *p* values were between 0.015 and 0.00001). About half of the characteristics increased whereas the other half decreased the risk of violence (Figure 4). Risk ratios were high with relatively rare characteristics, whereas risk ratios were low with frequent characteristics. Consequently, none of the critical characteristics on its own was able to predict violence.

Although the number of critical characteristics was relatively large (27 pro- and 25 anti-violence characteristics) one and the same individual possessed only a few of these. The largest number of coexisting pro- and anti-violence characteristics was 14 and 12, respectively, but such high numbers were present in one individual for each the violent and non-violent category. Typically, individuals expressed 4–5 pro- and/or anti-violence characteristics concomitantly. As the number of different combinations of characteristics was even larger than for the characteristics identified by Multiple Regression, we again used the number of characteristics to evaluate their predictive value at individual level.

**FIGURE 5:**
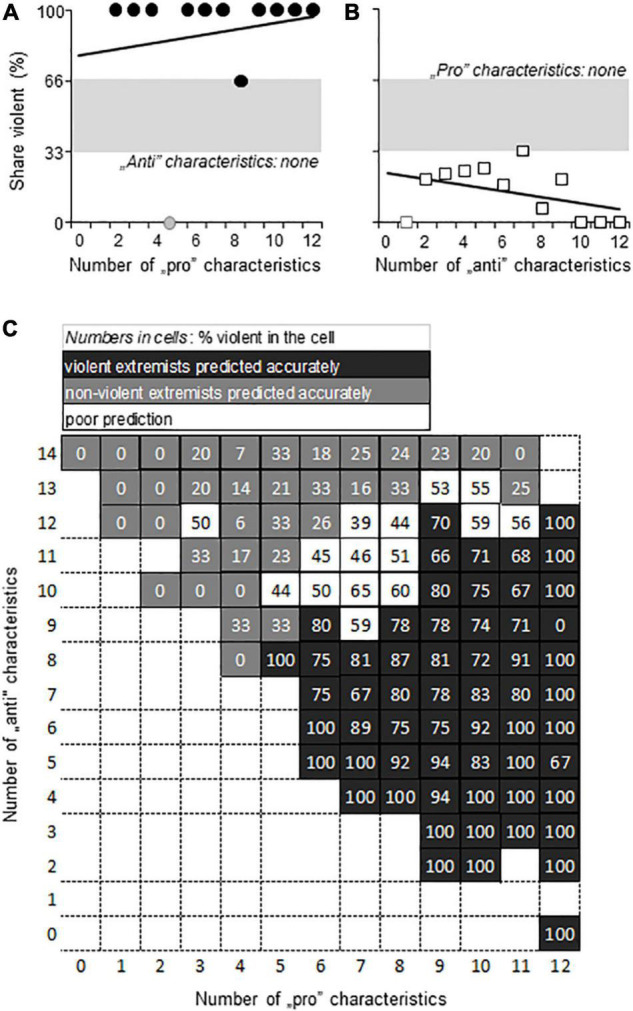
Violence prediction by the number of pro- and anti-violence characteristics identified by machine learning. **(A,B)** When the opposite characteristic was not present, pro-violence (“pro”) and anti-violence (“anti”) characteristics predicted violence with high accuracy. Note that sample sizes were low; symbols in gray indicate *N* = 1 for the given “pro”/”anti” characteristic combination. **(C)** The interactive dependence of violence on the relation between the number of pro-violence and anti-violence characteristics present in the individual. Gray in panels **(A–C)**, the area of chance prediction (34–65%, i.e., close to 50%).

If one of the characteristic types (e.g., pro- and antiviolence) was missing from the individual, the other characteristic type predicted violence with rather high precision (Figures 5A,B). However, such individuals were rare. Sample size for individuals with no anti-violence characteristics was 135, whereas for individuals without pro-violence characteristics was 26. Consequently, the prediction of violence depended on the interaction between pro- and anti-violence characteristics in 92% of the sample.

The representation of interactions between pro-violence characteristics, anti-violence characteristics, and the proportion of violent individuals in the cells of the interaction matrix (Figure 5C) was different from that seen with the characteristics identified by multiple Regression in two respects: (1) the pro- and anti-violence interaction matrix was considerably larger due to the larger number of critical characteristics; (2) predictions were more polarized. In most cases, the interaction reliably predicted the categorization of individuals into non-violent and violent groups (Figure 5C). Cells with low prediction power, i.e., where predicted group assignment was correct in 34–65% of cases only, were rare. Such indecisive predictions were seen in 14.7% of the cells. This was in sharp contrast with the characteristic matrix that was derived from Multiple Regression, where around one third of the cells provided indecisive predictions.

## Discussion

### Main Findings

We identified an array of critical variables that allowed the prediction of violence with an accuracy of over 85%. Machine learning performed better in this respect than the more conventional regression model. Within each critical variable, certain characteristics predicted violence and others predicted non-violence among radicalized individuals. Within the variable “Ideological Subcategory” for instance, the “Anti-gay” characteristic was the second strongest predictor of violence, whereas the “Animal Rights and Environmentalist” characteristic was the strongest predictor of non-violence. Most extremists displayed both pro- and antiviolence characteristics. These were displayed by extremists in many distinct combinations, which precluded their detailed analysis at this time. Yet the ratio of proviolent/antiviolent characteristics emerged as a strong predictor of violence and allowed the elaboration of a risk matrix that may be used to predict individual violence risk provided that enough critical characteristics are known for the individual. The characteristics of violent and non-violent extremists suggested that the two groups played different roles in terrorist attacks, suggesting the existence of work division among criminal extremists.

### Approaches and Variable Constellations

We found five earlier studies that employed the PIRUS database, which together identified 8 out of the 19 variables that predicted violence in our study (Jasko et al., 2017; LaFree et al., 2018, 2019; Becker, 2019; Jensen et al., 2020). Other factors recognized by us were indicated by studies on smaller databases (Berrebi, 2007; Altunbas and Thornton, 2011; Bartlett and Miller, 2012; Benmelech and Berrebi, 2007; Krueger, 2008; Meloy and Gill, 2016; Knight et al., 2017). These outlined, for instance, the roles of ethnicity, radical behaviors and beliefs, roles assumed by extremists within groups and economic status. The role of media was also revealed earlier (Cohen et al., 2014; Etudo, 2017; Youngblood, 2020). Taken together, this suggests that our study identified only 5 new variables and confirmed the importance of 14 (Table 6).

**TABLE 6:** Predictor variables in our and earlier studies.

Significant predictor this study	Becker, 2019	Jasko et al., 2017	Jensen et al., 2020	LaFree et al., 2018	LaFree et al., 2019	Other
Radical Behaviors						X
Radicalization Far Left				X		
Ideological Sub Category						
Broad Ethnicity		–				X
Radical Friend		X		X		
Religious Background						
Prison Radicalize					X	
Radicalization Islamist				X		
Group Membership	–					
Radical Family		X		X		
Role Group						X
Social Media						X
Convert						
Previous Criminal Activity	–	X	X	–		
Problematic Social Relations		–				
Radical Beliefs	X					X
Clique	X		X	–		
Media Radicalization						X
Social Stratum Adulthood		–				X

*This rough analysis shows that our predictor variables were identified earlier but in different studies, which precluded their direct comparison. X, significant predictor in earlier studies; –, non-significant predictor in earlier studies; Other, studies that used databases other then PIRUS; for references see text; white characters on black background, predictors not identified by earlier studies.*

Although there were important overlaps in our and earlier studies, we failed to confirm the predictive value of 15 variables that were found predictive in the above mentioned five studies that employed the PIRUS database. Importantly, the findings from these earlier studies were inconsistent themselves. For instance, younger age predicted violence in the studies by LaFree et al. (2018) and Jasko et al. (2017) but did not do so in the study by Becker (2019). On their turn, achieving aspirations and employment were negatively associated with violence in some studies (LaFree et al., 2018; Becker, 2019), but positively in others (Jasko et al., 2017). Similar examples are abundant.

These studies used the same PIRUS database but preselected variables according to their relevance for the hypothesis under scrutiny. As the hypotheses tested were different, the sets of selected variables showed little overlaps. One can hypothesize that their discrepant findings were primarily due to the constellation of variables under study, because multivariate analyses investigate interactions between variables. For instance, achieving aspirations correlated negatively with violence when considered together with employment (Becker, 2019) but positively when considered together with having a radical family and radical peers (Jasko et al., 2017). One can hypothesize that achieving aspirations in these two contexts results in different predictions regarding violence. As such, the predictive value of a variable can be changed by the constellation of variables investigated. This indirectly suggests that the more variables are considered the more equilibrated the result of the analysis becomes.

### Primary and Secondary Typologies

The development of extremist typologies is essential for counterterrorism, and several systems were developed to address this issue (Borum, 2003; Moghaddam, 2005; McCauley and Moskalenko, 2008; Aghabi et al., 2017). These were to a large extent based on the individual characteristics of terrorists. For instance, eight types were identified in a sample of 56 terrorists by studying 10 factors/variables (Jensen et al., 2020). None of the types presented with all the 10 factors; they were characterized by their unique combination. Not surprisingly, in our much larger sample and with a considerably larger number of factors considered, the number of characteristic-combinations was much larger, which prevented the development of a detailed typology. Nevertheless, our analysis did delineate two major types of extremists.

Typically, violent extremists came from criminal but not radical backgrounds and were converted and radicalized in later stages of their life, e.g., in early or mid-adulthood. They played minor roles in terrorist groups, sought training, and were radicalized largely by the social media. Also, they belonged to low social strata and had problematic social relations. These extremists may be termed executive/violent. By contrast, the non-violent but still extremists were characterized by a family tradition of radicalism, were less likely to have criminal backgrounds, belonged to higher social strata, played leadership roles in terrorist organizations, and instead of committing attacks, backed terrorism by supporting activities. This type may be termed organizing/non-violent.

A rough analysis of data suggests that both these major types are likely to be composed of subtypes. As compared to violent extremists fully, or mainly radicalized in prison, violent extremists who had no prison sentences or were fully radicalized before prison were less likely to be United States citizens (born or naturalized), had higher levels of education but poorer employment histories, belonged to higher social strata, and committed lesser crimes before radicalization. The same significant predictor of violence, particularly radicalization in prisons, also differentiated subgroups among non-violent extremists, but these subgroups were differentiated by a different set of variables. As compared to non-violent extremists radicalized in prison, those who were fully radicalized before prison were more likely to be students, had better employment histories, belonged to lower social strata, committed more severe crimes before radicalization, were less likely to have radical family friends, and were less likely to belong to a far-left ideology. It occurs that in some instances the subtypes of the two major types had opposite characteristics. For instance, prison radicalization in violent and non-violent extremists seemed to be associated with higher and lower, respectively, social status as compared to their peers belonging to the same type.

The analysis of such subtypes would stretch the boundaries of this study; yet the rough analysis presented above suggests that: (1) The critical variables identified in this study may be used to refine the current typologies and (2) the subtypes of the major types (the “executive/violent” and “organizing/non-violent” types) may be differentiated based on a different set of variables. As shown above for instance, violent extremists may be categorized into subgroups based on citizenship and prison radicalization, whereas the subtypes of non-violent extremists may be differentiated based education and prison radicalization.

### The Risk Matrix

Unsurprisingly, neither variable alone was able to predict violence accurately. In addition to the well-known complexity of the trait, this was also due to the nature of individual characteristics. These either had a large impact but were rare or the other way round were frequent, but their impact was low. For instance, the ideological subcategory “animal rights/environmentalist” predicted non-violence rather reliably yet only 7% of extremists belonged to this subcategory. By contrast, whites were frequent among radicals, but their risk ratio for violence was low (Figure 5). The power of interactions was shown by the fact that prediction accuracy increased by one order of magnitude when variables with significant contributions were considered together.

In this regard, our study showed that: (1) one and the same individual may show a mixture of pro- and anti-violence factors and (2) the number and ratio of these predicts either violence or non-violence with high accuracy when one prevails over the other. Putatively, the simple counting of pro- and anti-violence characteristics present in an individual and calculating their ratio or finding the place of the individual in the matrix shown in Figure 5C provides a good estimate of violence risk in the individual. Missing data may make such endeavors difficult, but one can assume that missingness is due to a certain extent to chance, and as such may not affect considerably the ratio of proviolence and antiviolence characteristics. As such, the risk matrix may be used by calculating ratios rather than counting characteristics. Many missing data makes analysis impossible, but this is a technical rather than a theoretical limitation.

### Earlier Hypotheses and the Findings With Machine Learning

As shown above, violent extremism was addressed from the point of view of communication science (Youngblood, 2020), criminology (LaFree et al., 2018, 2019), economics (Varaine, 2019), political science (Abrahms, 2012), social psychology (Jasko et al., 2017; Carson et al., 2019), and sociology (Becker, 2019; Ligon et al., 2019), to mention the most popular approaches. Our findings confirm all these but reveal a group of major factors that were not investigated earlier, suggest a “rank order” of the relative importance of theoretical assumptions, and show that various factors have different connotations in violent and non-violent extremists.

Over one third of critical characteristics relate to ideologies, religion, and ethnicity (Figure 4), which received little attention so far. Revealing such factors was the main reason for performing the analysis on the entire PIRUS sample, i.e., without breaking it down to ideological motivations. Noteworthy, the three often interconnected categories of factors may either decrease or increase the likelihood extremist violence, depending on their features. For instance, anti-gay, Sunni Islamist, and white supremacist extremism increase the likelihood of violence, whereas animal rights/environmentalist, and anti-abortion extremism decrease it (Figure 4). To our knowledge, such differences were not evidenced earlier by hypothesis-driven approaches, yet they appear as major factors of violence according to our machine learning approach.

Regarding the “rank order” of factors, criminological factors appear highly important as prison radicalization emerged as the strongest predictor of violent extremism. Previous violent crime also emerged as an important predictor, whereas never being in prison increased the likelihood of non-violence. Factors related to communication seemed to be the next most important category of factors as about one fifth of critical characteristics were related to communication. Interestingly, however, violent, and non-violent extremists seemed to be on the opposite side of the communication channel. While violent extremists were recipients who were strongly influenced by the social media, were converted, sought training, and were followers if members in groups, non-violent extremists were the deliverers of information by support, sharing beliefs, and leadership roles. Deprivation contributed to violent extremism as problematic social relationships and belonging to low social strata emerged as important predictors of violence. Extremism *per se*, however, did not seem to be associated with deprivation as non-violent extremists usually belonged to high social strata. Finally, social learning from family and friends played surprisingly little role as violent extremists usually came from non-extremist backgrounds. On the other hand, non-violent extremists usually came from backgrounds favoring legal or non-violent illegal extremism. As such, non-violent rather than violent extremism seemed to be associated with social learning.

These findings and conclusion naturally need further experimental support by similar studies made on different terrorist databases. Such studies may clarify the generalizability of conclusions, which at present are tightly bound to the particularities of one single database. Furthermore, the machine learning approach should also be employed to investigate separately extremists belonging to different ideologies. Such studies may reveal ideology-specific risk factors, and risk matrices.

### Limitations

The largest limitations of our analysis were the missing data, which, however, cannot be amended presently. All studies that use large datasets are faced with this problem (Safer-Lichtenstein et al., 2017; Knight and Keatley, 2020). Several methods were proposed to address this issue. Jasko et al. (2017) for instance, analyzed both the original PIRUS dataset and the same after replacing missing data by multiple imputation. LaFree et al. (2018) compared four different methods to substitute missing data—including mean substitution and multiple imputations—and concluded that they were equivalent. In sharp contrast, Safer-Lichtenstein et al. (2017) found that the results of analyses greatly depend on the amount of missing data. In our study, mean substitution worsened whereas multiple imputation did not affect the outcome of analyses.

Another limitation was the inclusion of all ideological backgrounds despite that Far Right, Far Left, Islamist, and Single-Issue extremists differ from each other in multiple ways (Jensen et al., 2015; Al-Zewairi and Naymat, 2017; Freilich et al., 2018). We studied all extremists together because this allowed the comparison of ideologies within the same analysis. This showed that among ideologies anti-gay and Sunni Islam were the most likely indicators for violence. These were followed by Islam ideology in general, and Far Right ideology, whereas certain ideologies, for instance, Far Left, Anti-abortion, Single-issue, Animal Rights and Environment ideologies were protective against violence. Naturally, this does not decrease the potential value of the separate analysis of different ideologies. This, however, remains for forthcoming studies.

The individual combinations of various risk and protective factors was insufficiently addressed in this study. The reason was that we aimed at comparing ideological backgrounds within the same analysis, which inherently made the sample heterogenous. Separate analyses along ideological backgrounds would likely result in more homogenous samples, which will enable us to analyze individual combinations of risk factors in future studies.

Finally, the database was probably not free of errors, which cannot be checked presently. However, the database was compiled by a restricted number of researchers based on a unitary system of carefully established principles. Our findings need of course crosschecking with a similar study to be done on other databases.

## Conclusion

The XGBoost algorithm delineated a set of 19 variables that covered in total 53 individual characteristics based on which the risk of extremist violence could be predicted with more than 85% accuracy. This is in the upper region of the accuracies reported for a variety of algorithms that evaluated violence-unrelated features of radicalization (see paragraph 9 in Introduction). We submitted the critical factors and characteristics to a deeper analysis and based on this we suggest that machine learning algorithms may be successfully used to evaluate the relative weight of, and relationship between variables and characteristics, and may be used to improve both terrorist typologies and risk assessment guidelines.

We suggest that the risk matrix developed here may already be tested as a risk assessment tool for violent extremism. More importantly, however, this study shows the power of machine learning in studying the complex relationships between various factors that endanger violence in extremists, which confers both theoretical and practical perspectives to this approach.

## Data Availability Statement

Publicly available datasets were analyzed in this study. This data can be found here: https://www.start.umd.edu/profiles-individual-radicalization-united-states-pirus-keshif.

## Author Contributions

KI conceptualized the study and performed the statistical analysis of machine learning. JH performed the statistical analysis of multiple regression. Both authors contributed to the article and approved the submitted version.

## Conflict of Interest

The authors declare that the research was conducted in the absence of any commercial or financial relationships that could be construed as a potential conflict of interest.

## Publisher’s Note

All claims expressed in this article are solely those of the authors and do not necessarily represent those of their affiliated organizations, or those of the publisher, the editors and the reviewers. Any product that may be evaluated in this article, or claim that may be made by its manufacturer, is not guaranteed or endorsed by the publisher.

## Footnotes

1
http://www.start.umd.edu/pirus
